# Impact of visceral pleural invasion on the association of extent of lymphadenectomy and survival in stage I non‐small cell lung cancer

**DOI:** 10.1002/cam4.1990

**Published:** 2019-02-01

**Authors:** Yang Wo, Yandong Zhao, Tong Qiu, Shicheng Li, Yuanyong Wang, Tong Lu, Yi Qin, Guisong Song, Shuncheng Miao, Xiao Sun, Ao Liu, Dezhi Kong, Yanting Dong, Xiaoliang Leng, Wenxing Du, Wenjie Jiao

**Affiliations:** ^1^ Department of Thoracic Surgery Affiliated Hospital of Qingdao University Qingdao China

**Keywords:** lung cancer, lymph node, survival, visceral pleural invasion

## Abstract

Visceral pleural invasion (VPI) has been identified as an adverse prognostic factor for non‐small cell lung cancer (NSCLC). Accurate nodal staging for NSCLC correlates with improved survival, but it is unclear whether tumors with VPI require a more extensive lymph nodes (LNs) dissection to optimize survival. We aimed to evaluate the impact of VPI status on the optimal extent of LNs dissection in stage I NSCLC, using the Surveillance, Epidemiology, and End Results (SEER) database. We identified 9297 surgically treated T1‐2aN0M0 NSCLC patients with at least one examined LNs. Propensity score matching was conducted to balance the baseline clinicopathologic characteristics between the VPI group and non‐VPI group. Log‐rank tests along with Cox proportional hazards regression methods were performed to evaluate the impact of extent of LNs dissection on survival. VPI was correlated with a significant worse survival, but there was no significant difference in survival rate between PL1 and PL2. Patients who underwent sublobectomy had slightly decreased survival than those who underwent lobectomy. Pathologic LNs examination was significantly correlated with survival. Examination of 7‐8 LNs and 14‐16 LNs conferred the lowest hazard ratio for T1‐sized/non‐VPI tumors (stage IA) and T1‐sized/VPI tumors (stage IB), respectively. The optimal extent of LNs dissection varied by VPI status, with T1‐sized/VPI tumors (stage IB) requiring a more extensive LNs dissection than T1‐sized/non‐VPI tumors (stage IA). These results might provide guidelines for surgical procedure in early stage NSCLC.

## 1 INTRODUCTION

Lung cancer has been identified as the leading cause of cancer death for decades with a high incidence worldwide.^1^ Approximately 85% of lung cancer patients are diagnosed with non‐small cell lung cancer (NSCLC). Surgical resection, whenever possible, is generally the preferred treatment modality for early stage NSCLC.^2^

Visceral pleural invasion (VPI) was determined as a negative prognosticator in NSCLC and was first incorporated into the fifth edition tumor, node, metastasis (TNM) staging criteria in 1997.^3^ The International Association for the Study of Lung Cancer (IASLC) recommended the classification of the status of VPI as follows: PL0, tumor grows within the parenchyma or does not completely penetrate the elastic layer; PL1, tumor extends beyond the elastic layer; PL2, tumor invades into the surface of the visceral pleura.^2^, ^4^ To sum up, PL0 indicates no evidence of VPI, while PL1 and PL2 both represent the invasion of visceral pleural. In the seventh and eighth edition of TNM staging system, VPI has been identified as a non‐size‐based T2 factor, upstaging tumors ≤3 cm to T2a. Previous studies demonstrated that patients with VPI correlated with a higher incidence of pleural effusion, mediastinal nodal metastasis and postoperative recurrence, emphasizing the significance of improving treatment strategies.^5^, ^6^, ^7^

Adequate lymph nodes (LNs) assessment is associated with favorable prognosis and plays a crucial part in the accurate staging of NSCLC. Previous studies illustrated the phenomenon that survival rate improved as more LNs were dissected in surgically resected NSCLC.^8^, ^9^, ^10^, ^11^ For example, Liang and colleagues studied the relationship between the examined LNs count and survival in NSCLC and identified 16 examined LNs as the optimal cut‐off point for evaluating the quality and thoroughness of LNs dissection.^8^ Samayoa et al. confirmed that survival of surgically resected node‐negative NSCLC patients was closely associated with the thoroughness of lymphadenectomy and recommended that at least 10 LNs should be examined.^10^ As far as we know, none of those previous studies explored the impact of the status of VPI on the correlation between the extent of lymphadenectomy and survival in stage I NSCLC. The goal of our present study was to determine whether patients with T1‐sized/VPI tumors (stage IB) required more extensive LNs dissection to optimize survival than those with T1‐sized/non‐VPI tumors (stage IA), using a large population‐based database.

## 2 METHOD

### 2.1 Data collection

Research data were extracted from the Surveillance, Epidemiology, and End Results (SEER) program November 2017 update via SEER*Stat software (National Cancer Institute, Bethesda, MD). In the past decades, SEER program progressively expanded from the previous nine registries to the current 18 registries covering approximately 28% of American population. From SEER database, all cases with microscopically confirmed NSCLC were selected. Patients who underwent pneumonectomy and those who underwent preoperative radiotherapy were excluded from this study. Histologic grade was reclassified as low grade (well differentiated and moderately differentiated) or high grade (poorly differentiated and undifferentiated). VPI status has been brought into SEER database since 2010 based on the data term, cs site‐specific factor 2. Thus, our study only included the patients who were diagnosed between 2010 and 2015. Furthermore, since the staging criteria of our study population were based on the seventh edition TNM staging criteria, patients were then reclassified according to the eighth edition TNM staging criteria. We only identified cases with pathologic T1‐2aN0M0 NSCLC owing to the possibility of invading adjacent structure or organ in T3‐4 stage. Our study also eliminated patients with T1‐sized tumors, but upstaged to T2a due to hilar atelectasis or obstructive pneumonia. Baseline demographics, cancer characteristics, and survival data were collected including ethnicity, marital status, sex, age of diagnosis, tumor grade and size, therapeutic method, number of LNs examined, death classification, and survival months. Since none of the protocols involved raw data collection and the patient data were anonymized and openly accessible, Institutional Review Board approval was not required.

### 2.2 Statistical analysis

All statistical analysis was conducted on R version 3.5.1 (R foundation for statistical computing, Vienna, Austria) and SPSS version 22 (IBM Corp., Armonk, NY). To reduce disparities of baseline characteristics between the VPI group and non‐VPI group, we therefore conducted propensity score matching (PSM). Baseline characteristics (ethnicity, age, sex, tumor size, marital status, histologic type and grade) were incorporated in the propensity score analysis. *MatchIt* package in R was used to match patients in two groups by propensity scores with a 1:1 nearest neighbor matching. Lung cancer‐specific survival (LCSS), defined as the survival time from lung cancer diagnosis to death specific to lung cancer‐related death, was the primary outcome variable and was estimated with Kaplan‐Meier analyses. The difference in survival curves was determined by Log‐rank tests. Continuous variables were presented as mean ± SD and were compared using the Student *t* test, while categorical variables were expressed as frequency (percentage) and were measured with the Pearson chi‐squared test. Cox regression analyses were conducted to evaluate the impact of the number of examined LNs on survival, adjusted for other potential confounding clinicopathological factors. The optimal number of examined LNs was identified by analyzing the trend in hazard ratios (HR) calculated by multivariate Cox regression model, and the turning point in the HR curve was exactly the optimal examined LNs count. All statistical analyses were two‐sided, and a *P*‐value ≤0.05 was considered statistically significant.

## 3 RESULT

**Figure 1:**
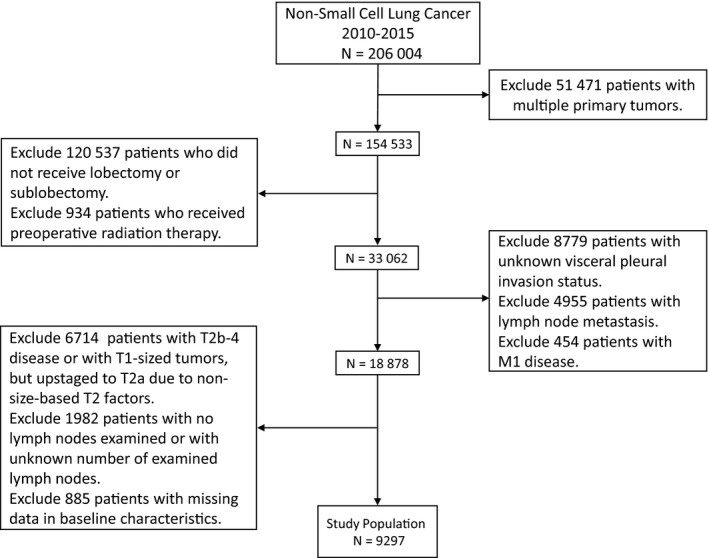
Study selection map

**Figure 2:**
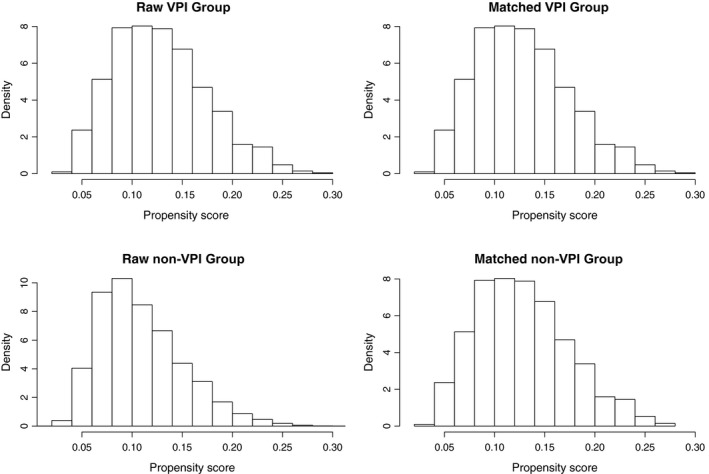
Histograms demonstrating the distribution of propensity score before and after matching. VPI, visceral pleural invasion

Our study finally identified 9297 NSCLC patients who met the inclusion criteria. Figure 1 shows the data collection criteria of this study. In total, 1034 cases were diagnosed with VPI, including 586 patients with PL1 and 448 patients with PL2, while PL0 was identified in 8263 patients. Significant discrepancies in age, histologic type, race distribution, histologic grade, tumor size, and treatment modality were observed between the two cohorts (Table 1). Specifically, patients diagnosed with VPI were more likely to be older, to be diagnosed with adenocarcinoma, to have poor differentiation and larger tumors, and to complete adjuvant radiation, which indicated the imbalance in the baseline clinicopathological features between the unmatched groups. Therefore, we conducted PSM and 1034 pairs stratified by the status of VPI were successfully matched. The distribution of propensity scores before and after matching was shown in Figure 2.

**Table 1:** Patients characteristics before and after matching

Variable	Before matching			After matching		
	Non‐VPI (n = 8263)	VPI (n = 1034)	*P‐*value	Non‐VPI (n = 1034)	VPI (n = 1034)	*P*‐value
Age	67.3 ± 9.1	68.0 ± 9.2	0.021	68.1 ± 9.2	68.0 ± 9.2	0.782
Histologic type			<0.001			0.969
Adenocarcinoma	6181 (74.8)	834 (80.7)		836 (80.9)	834 (80.7)	
Squamous cell carcinoma	2014 (24.4)	191 (18.4)		190 (18.3)	191 (18.4)	
Other	68 (0.8)	9 (0.9)		8 (0.8)	9 (0.9)	
Sex			0.205			0.332
Female	4742 (57.4)	572 (55.3)		550 (53.2)	572 (55.3)	
Male	3521 (42.6)	462 (44.7)		484 (46.8)	462 (44.7)	
Race			0.035			0.782
Black	689 (8.3)	103 (10.0)		103 (10.0)	103 (10.0)	
Other	640 (7.8)	96 (9.3)		87 (8.4)	96 (9.3)	
White	6934 (83.9)	835 (80.7)		844 (81.6)	835 (80.7)	
Marital status			0.574			0.687
Married	4791 (58.0)	609 (58.9)		618 (59.8)	609 (58.9)	
Unmarried	3472 (42.0)	425 (41.1)		416 (40.2)	425 (41.1)	
Grade			<0.001			0.442
Low grade	6224 (75.3)	717 (69.3)		733 (70.9)	717 (69.3)	
High grade	2039 (24.7)	317 (30.7)		301 (29.1)	317 (30.7)	
Tumor size(mm)	18.7 ± 6.2	20.9 ± 5.7	<0.001	20.9 ± 5.9	20.9 ± 5.7	0.748
Surgery			0.534			0.295
Lobectomy	6825 (82.6)	846 (81.8)		864 (83.6)	846 (81.8)	
Sublobectomy	1438 (17.4)	188 (18.2)		170 (16.4)	188 (18.2)	
Number of examined LNs	9.4 ± 7.5	9.3 ± 7.5	0.765	9.4 ± 7.5	9.3 ± 7.5	0.711
Adjuvant radiation			0.001			0.015
No	8156 (98.7)	1007 (97.4)		1022 (98.8)	1007 (97.4)	
Yes	107 (1.3)	27 (2.6)		12 (1.2)	27 (2.6)	

VPI, visceral pleural invasion; LNs, lymph nodes.

**Figure 3:**
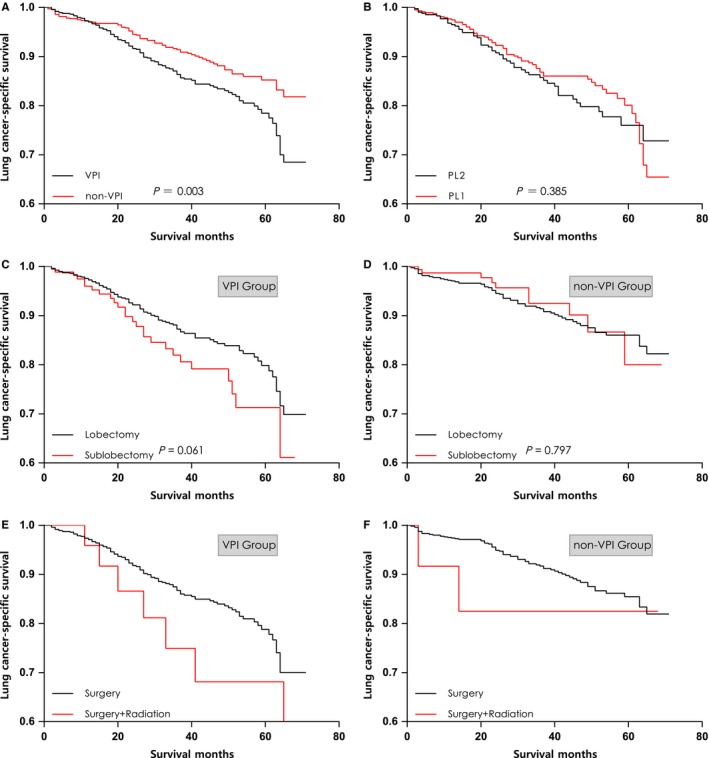
Survival curves stratified by (A) presence or absence of VPI, (B) depth of VPI, (C) resection scope in VPI group, (D) resection scope in non‐VPI group, (E) treatment modality in VPI group, and (F) treatment modality in non‐VPI group. VPI, visceral pleural invasion

The Kaplan‐Meier curves revealed that patients with VPI had a decreased survival compared with those in the non‐VPI group (5‐year LCSS: 78.2% vs 85.1%; *P *=* *0.003; Figure 3A). Nevertheless, there was no significant difference in LCSS between PL1 and PL2. (5‐year LCSS 80.1% vs 75.7%; *P* = 0.385; Figure 3B). In regard to the treatment modality, we further analyzed the prognostic value of surgical extent and adjuvant radiotherapy in each group. Patients who underwent sublobectomy had slightly decreased survival than those who underwent lobectomy, but the difference is not statistically significant in either VPI group (5‐year LCSS 71.3% vs 79.8%; *P *=* *0.061; Figure 3C) or non‐VPI group (5‐year LCSS 79.8% vs 86.0%; *P *=* *0.797; Figure 3D). Unexpectedly, patients who received adjuvant radiotherapy had worse LCSS than those who underwent surgery alone in both VPI group (Figure 3E) and non‐VPI group (Figure 3F). Owing to limited number of cases in radiotherapy group and short follow‐up time, we could not precisely evaluate the therapeutic benefit of adjuvant radiotherapy.

**Figure 4:**
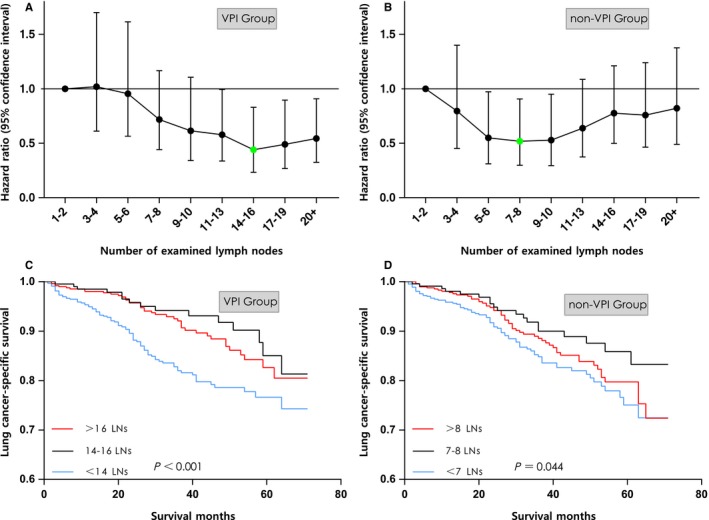
The variance of Hazard Ratio with 95% confidence interval among different examined LNs count in VPI group (A) and non‐VPI group (B), with the green dot indicating the optimal cut‐point. Survival curves of patients stratified by the examined LNs count in VPI group (C) and non‐VPI group (D). VPI, visceral pleural invasion; LNs, lymph nodes

Cox regression models were conducted to estimate the adjusted HR depending on the examined LNs count. In the VPI group, examination of at least 11 LNs were significantly associated with survival benefit and patients with 14‐16 examined LNs had the lowest risk of death (HR, 0.439; 95% confidence intervals [CI], 0.233‐0.830; *P *=* *0.011; Figure 4A). By contrast, in patients without VPI, the risk of death sequentially decreased as more LNs were examined until a maximal survival benefit was reached with examination of 7‐8 LNs (HR, 0.519; 95% CI, 0.297‐0.906; *P *=* *0.021); however, the sequential improvement in survival was not statistically significant after examination of more than 10 LNs (Figure 4B). Kaplan‐Meier curves also demonstrated superior survival benefits in VPI group with 14‐16 retrieved LNs (Figure 4C) and non‐VPI group with 7‐8 retrieved LNs (Figure 4D). In the multivariate analysis, homogeneous prognostic factors for the VPI group and non‐VPI group included examined LNs count, tumor size, and tumor grade, whereas age was solely significant in the VPI group (Table 2, 3).

**Table 2:** Cox regression analysis of prognostic factors in T1‐sized/VPI tumors (stage IB)

Variable	Univariate		Multivariate	
	HR (95% CI)	*P*‐value	HR (95% CI)	*P*‐value
Age	1.031 (1.011‐1.052)	0.003	1.025 (1.004‐1.046)	0.021
Histologic type				
Adenocarcinoma	Reference			
Squamous cell carcinoma	1.471 (0.973‐2.225)	0.067		
Other	2.098 (0.519‐8.595)	0.296		
Sex				
Female	Reference			
Male	1.171 (0.820‐1.675)	0.385		
Race				
Black	Reference			
Other	1.257 (0.522‐3.022)	0.610		
White	1.223 (0.593‐2.342)	0.544		
Marital status				
Married	Reference			
Unmarried	1.335 (0.934‐1.909)	0.113		
Grade				
Low grade	Reference		Reference	
High grade	1.812 (1.261‐2.603)	0.001	1.773 (1.226‐2.565)	0.002
Tumor size(mm)	1.040 (1.007‐1.074)	0.017	1.031 (0.998‐1.065)	0.070
Surgery				
Lobectomy	Reference			
Sublobectomy	1.500 (0.977‐2.303)	0.063		
Examined LNs count				
1‐2	Reference		Reference	
3‐4	0.980 (0.588‐1.634)	0.938	1.019 (0.611‐1.701)	0.942
5‐6	0.921 (0.545‐1.555)	0.757	0.955 (0.565‐1.615)	0.865
7‐8	0.672 (0.425‐1.108)	0.153	0.708 (0.444‐1.174)	0.166
9‐10	0.608 (0.340‐1.090)	0.095	0.614 (0.341‐1.106)	0.104
11‐13	0.562 (0.328‐0.965)	0.037	0.579 (0.337‐0.994)	0.048
14‐16	0.428 (0.241‐0.772)	0.004	0.439 (0.233‐0.830)	0.011
17‐19	0.471 (0.258‐0.861)	0.006	0.490 (0.268‐0.896)	0.021
≥20	0.505 (0.308‐0.909)	0.031	0.524 (0.331‐0.924)	0.041
Extend of VPI				
PL1	Reference			
PL2	1.172 (0.819‐1.676)	0.386		
Adjuvant radiation				
No	Reference			
Yes	1.917 (0.893‐4.112)	0.095		

HR, hazard ratio; CI, confidence interval; VPI, visceral pleural invasion; LNs, lymph nodes.

**Table 3:** Cox regression analysis of prognostic factors in T1‐sized/non‐VPI tumors (stage IA)

Variable	Univariate		Multivariate	
	HR (95% CI)	*P*‐value	HR (95% CI)	*P*‐value
Age	1.028 (0.976‐1.072)	0.104		
Histologic type				
Adenocarcinoma	Reference			
Squamous cell carcinoma	1.610 (1.369‐1.893)	0.094		
Other	1.415 (0.203‐8.847)	0.346		
Sex				
Female	Reference			
Male	1.055 (0.680‐1.637)	0.811		
Race				
Black	Reference			
Other	0.234 (0.052‐1.046)	0.057		
White	0.730 (0.395‐1.352)	0.317		
Marital status				
Married	Reference			
Unmarried	1.178 (0.759‐1.828)	0.466		
Grade				
Low grade	Reference		Reference	
High grade	1.756 (1.127‐2.737)	0.013	1.728 (1.105‐2.701)	0.016
Tumor size (mm)	1.053 (1.013‐1.094)	0.009	1.058 (1.018‐1.100)	0.004
Surgery				
Lobectomy	Reference			
Sublobectomy	0.920 (0.486‐1.740)	0.797		
Examined LNs count				
1‐2	Reference		Reference	
3‐4	0.780 (0.443‐1.372)	0.389	0.796 (0.452‐1.400)	0.427
5‐6	0.513 (0.285‐0.921)	0.025	0.556 (0.309‐0.968)	0.042
7‐8	0.492 (0.282‐0.858)	0.012	0.519 (0.297‐0.906)	0.021
9‐10	0.505 (0.281‐0.907)	0.022	0.529 (0.294‐0.950)	0.033
11‐13	0.623 (0.366‐1.061)	0.081	0.638 (0.374‐1.086)	0.098
14‐16	0.702 (0.416‐1.151)	0.164	0.716 (0.522‐1.254)	0.194
17‐19	0.716 (0.439‐1.169)	0.182	0.759 (0.464‐1.240)	0.271
≥20	0.805 (0.479‐1.354)	0.414	0.827 (0.488‐1.371)	0.476
Adjuvant radiation				
No	Reference			
Yes	1.751 (0.430‐7.128)	0.434		

HR, hazard ratio; CI, confidence interval; VPI, visceral pleural invasion; LNs, lymph nodes.

## 4 DISCUSSION

Although surgical resection confers significant therapeutic benefit and remains the first choice of treatment, the optimal extent of LNs evaluation for early stage NSCLC patients without any signs of LNs metastasis or distant disease is still under debate. Consistent with previous research,^8^, ^9^, ^10^, ^11^, ^12^, ^13^, ^14^ our study revealed the association of the examined LNs count with survival. Furthermore, we also have confirmed that the correlation between the extensiveness of LNs dissection and prognosis is dependent on VPI status, with T1‐sized/VPI tumors (stage IB) requiring a more extensive LNs dissection (14‐16 LNs) while T1‐sized/non‐VPI tumors (stage IA) requiring a less extensive LNs dissection (7‐8 LNs).

Since previous research which also investigated the optimal examined LNs count did not consider tumor size and VPI status, their findings were not applicable for all‐comers and thoracic surgeons could not handle all patients in the same way. The interaction of VPI status and the optimal examined LNs count could provide guidance on the management and treatment of early stage NSCLC. Deng et al. comprehensively investigated predictors of VPI in patients with T1‐sized NSCLC and identified older age, adenocarcinoma, poor differentiation, pleural indentation, and shorter distance from tumor edge to visceral pleural as significant risk factors of VPI.^15^ In clinic, when preoperative general examination revealed risk factors of VPI or intraoperative exploration suspected the diagnosis of VPI, surgeons were advised to perform a more extensive lymphadenectomy to optimize the survival benefit.

The impact of the examined LNs count on survival could be attributed to several underlying reasons. On the one hand, logic suggested that removal of more LNs would increase the likelihood of detecting metastatic LNs and contribute to improved nodal staging accuracy. A less extensive lymphadenectomy, however, conferred increased risk of omitting undiscovered metastatic LNs. Some patients with fewer examined LNs and declared N0 disease may actually have node‐positive disease and this group of patients was less likely to receive proper adjuvant therapy due to understaging (e.g., misdiagnosis of patients with nodal metastasis as N0 disease). Considering patients in VPI group had a higher incidence of LNs metastasis,^5^, ^16^, ^17^ we hence speculated that a greater number of undiscovered metastatic LNs existed in VPI group with declared N0 disease. Thus, more extensive LNs examination was recommended for patients with VPI to improve staging accuracy and the chance of cure. On the other hand, more extensive LNs examination reflected not only surgeon's proficiency in LNs dissection but also pathologist's technique in LNs examination, and thus could potentially affect surgical outcomes. As described in a previous study, exquisite operative technique and remarkable patient care provided by high‐volume medical center may improve prognosis of early stage NSCLC patients.^18^ In addition, as filters for cancer cells and foreign bodies, LNs are critical component of human immune system. The negative LNs count also might reflect the intensity of anticancer immune response, which would influence survival.^19^

The negative prognostic effect of VPI in surgically resected NSCLC has been well‐defined. Nevertheless, the prognostic significance of the depth of VPI (PL1 vs PL2) was still under debate. In this study, VPI was correlated with a significant decreased survival, but there was no significant difference in survival rate between PL1 and PL2. Consistent with our results, Adachi et al. demonstrated that presence of VPI, instead of the depth, was correlated with postoperative survival.^5^ In contrast, hung and colleagues identified PL2 as an indicator of worse survival and frequent recurrence in node‐negative NSCLC.^6^ A recent meta‐analysis conducted by Wang et al. also confirmed that the survival of PL1 patients was superior to that of PL2 patients.^17^ Although the eighth TNM staging system did not incorporate the extent of VPI into T descriptor, the IASLC did indicate that PL2 had a wore prognosis.^20^ Therefore, additional prospective studies were warranted to clarify the prognostic significance of the depth of VPI in early stage NSCLC patients.

The optimal resection scope of stage I NSCLC is still under debate and the optimal treatment modality for patients with VPI remains unclear. Moon and colleagues studied the surgical outcomes of 271 NSCLC patients with angiolymphatic or visceral pleural invasion and revealed that survival rate did not differ significantly by surgical extent, but their study did not distinguish VPI from angiolymphatic invasion and was based on a small sample size.^21^ A recent PSM study designed by Subramanian et al. identified that sublobar resection groups could acquire identical survival benefit as lobectomy groups in stage I NSCLC.^22^ On the contrary, two published meta‐analysis declared a significant worse survival in limited resection group.^23^, ^24^ In this study, although the extent of resection did not significantly correlate with LCSS in both VPI group and non‐VPI group, patients who underwent sublobectomy had slightly decreased survival than those who underwent lobectomy. And at the same time, it is worth noting that the survival curves stratified by the extent of resection almost clustered together in T1‐sized/non‐VPI tumors (stage IA), but well‐separated in T1‐sized/VPI tumors (stage IB) without superposition. Besides, in Cox regression models, limited resection of T1‐sized/VPI tumors (stage IB) correlated with increased mortality risk (HR = 1.500), while the HR for T1‐sized/non‐VPI tumors (stage IA) undergoing limited resection was close to 1 (HR = 0.920). Moreover, the *P*‐value of log‐rank test (*P *=* *0.061) and univariate Cox regression analysis (*P *=* *0.063) concerning the association of surgical approach with survival in VPI group was close to the threshold (0.05). Nevertheless, the limited number of cases in the sublobar resection group and relatively short follow‐up time of our study cohort restricted us to perform detailed analysis of optimal extent of surgical resection for stage I NSCLC stratified by tumor size and VPI status. Given the above analysis, we recommend active follow‐up for T1‐sized/VPI tumors (stage IB) after sublobar resection. The benefit of performing lobectomy in the VPI group could be attributed to the greater chance of radical dissection of involved visceral pleural and lymphatic vessels. Previous studies showed that adjuvant radiation failed to confer survival benefit for early stage NSCLC, but none of those studies incorporated VPI status.^25^, ^26^ In this study, patients with VPI had a higher likelihood of receiving adjuvant radiotherapy. However, the therapeutic benefit of adjuvant radiotherapy could not be precisely measured owing to relatively small number of radiated patients.

This population‐based study also has several limitations, including constraint of SEER database and the retrospective nature. First, the protocol for dissecting and examination of LNs may vary among different medical centers and the expertise of surgeons and pathologists was unknown, which could affect the quality of LNs dissection. Second, although the SEER registry provided information about examined LNs count, the number of examined LNs station, which is free from the risk of being confounded by fragmentation of LNs, was not recorded. However, in spite of the risk of being confounded by the fragmentation, the examined LNs count remains an appropriate surrogate for the thoroughness in LNs examination. Moreover, data on comorbidities, complications, chemotherapy, pulmonary function, and surgical margin were not available, which could cause potential bias.

In conclusion, this study demonstrates that the extent of LNs examination required to optimize survival differs based on VPI status in stage I NSCLC, with T1‐sized/VPI tumors (stage IB) requiring a more extensive LNs examination than T1‐sized/non‐VPI tumors (stage IA).

## CONFLICTS OF INTEREST

The authors declare that there are no conflicts of interest.
